# How to Handle Co-authorship When Not Everyone’s Research Contributions Make It into the Paper

**DOI:** 10.1007/s11948-021-00303-y

**Published:** 2021-04-12

**Authors:** Gert Helgesson, Zubin Master, William Bülow

**Affiliations:** 1 Stockholm Centre for Healthcare Ethics (CHE), Department of Learning, Informatics, Management and Ethics, Karolinska Institutet, Stockholm, Sweden; 2 Biomedical Ethics Research Program and Center for Regenerative Medicine, Mayo Clinic, Rochester, MN USA; 3 Department of Philosophy, Stockholm University, Stockholm, Sweden

**Keywords:** Authorship, Authorship criteria, Ethics, Negative results, Substantial contribution

## Abstract

While much of the scholarly work on ethics relating to academic authorship examines the fair distribution of authorship credit, none has yet examined situations where a researcher contributes significantly to the project, but whose contributions do not make it into the final manuscript. Such a scenario is commonplace in collaborative research settings in many disciplines and may occur for a number of reasons, such as excluding research in order to provide the paper with a clearer focus, tell a particular story, or exclude negative results that do not fit the hypothesis. Our concern in this paper is less about the reasons for including or excluding data from a paper and more about distributing credit in this type of scenario. In particular, we argue that the notion ‘substantial contribution’, which is part of the International Committee of Medical Journal Editors (ICMJE) authorship criteria, is ambiguous and that we should ask whether it concerns what ends up in the paper or what is a substantial contribution to the research process leading up to the paper. We then argue, based on the principles of fairness, due credit, and ensuring transparency and accountability in research, that the latter interpretation is more plausible from a research ethics point of view. We conclude that the ICMJE and other organizations interested in authorship and publication ethics should consider including guidance on authorship attribution in situations where researchers contribute significantly to the research process leading up to a specific paper, but where their contribution is finally omitted.

## Introduction

Research typically proceeds in less predictable ways than we like to acknowledge. While a scientific ideal is that every part of a study is well considered and planned beforehand, and the research process thereafter mainly consists in performing according to protocol, a typical experience from the field is that such description is far from the truth. Planning and execution of plans are rarely that straightforward. To the contrary, many decisions are made along the way regarding both data collection and analysis: new experiments, comparisons, interviews, and surveys may be decided as the work proceeds, and additional analyses may be added to the ones originally decided upon. Sometimes these changes are driven by peer review. Some of the research contributions eventually pass critical scrutiny and make it into the paper, while others for one reason or another end up in the waste bin or are shelved for possible future use.

There may be positive as well as negative things to say about such practice in relation to the philosophy of science and meta science, relating to curiosity and creativity on the one hand and hypothesis testing and reproducibility issues on the other. Furthermore, some of the choices made regarding what results get included in papers may be objectionable from a research ethics perspective, such as excluding ‘negative results’ because they contradict the main results of the paper or are considered unworthy of publication, hence contributing to the positive publication bias (Chalmers et al., 1990; Connor, 2008; Dirnagl & Lauritzen, 2010). Other exclusions may be fully acceptable, based on estimations of relevance and the consideration that you cannot include everything in the paper if it is to be readable.

The present paper does not deal with what exclusions of research results are acceptable or not, but with a related issue, namely how to handle authorship attributions in papers where plans change along the way, so that some of the results derived are not included in the final version of the paper. This topic is a special case of the larger topic of who should be included as authors on research papers, which involves ethical aspects like appropriate allocation of credit in research (and, hence, fairness), transparency and accountability (Shamoo & Resnik, 2009). Given the frequency of authorship disagreements (Marušić et al., 2011; Nylenna et al., 2014; Okonta & Rossouw, 2013), this analysis is important also because good authorship practices serve to foster positive team dynamics and collaboration and are less likely to lead to authorship disputes, which could impact interpersonal and professional relationships and possibly lead to subsequent misbehaviors (Smith et al., 2020; Tijdink et al., 2016).

The International Committee of Medical Journal Editors (ICMJE) has produced the most widely acknowledged authorship recommendations, which serve as guidance for the biomedical sciences among other areas of scholarship (ICMJE, 2019). The recommendations provide a set of criteria that are jointly necessary and sufficient in order to determine authorship (see Box 1). While these authorship criteria have met their due share of criticism (Laflin et al. 2005; Osborne & Holland, 2009; Puljak & Sambunjak, 2000; Smith et al. 2014), we find them reasonable, although not flawless (see e.g. Helgesson & Eriksson, 2018; Helgesson et al 2019). We therefore treat the ICMJE criteria as the starting point for our discussion of whether granting authorship to someone can be justified even when that person’s specific contributions do not make it into the final version of the manuscript. However, the problem we have identified, having to do with the first criterion–that authors should have made a ‘substantial contribution’ to the work–is not clearly addressed by the ICMJE recommendations. In fact, the problem is overlooked by most suggestions for how to think about the allocation of authorship in co-researched papers (see e.g., Shamoo & Resnik, 2009; Hansson, 2017; Moffatt, 2018).

**Box 1:** ICMJE Authorship recommendations

The International Committee of Medical Journal Editors (ICMJE) recommends that authorship be given to researchers who have contributed substantially and have satisfied the following 4 criteria:
Substantial contributions to the conception or design of the work; or the acquisition, analysis, or interpretation of data for the work; AND Drafting the work or revising it critically for important intellectual content; AND Final approval of the version to be published; AND Agreement to be accountable for all aspects of the work in ensuring that questions related to the accuracy or integrity of any part of the work are appropriately investigated and resolved.
The ICMJE explain that the four criteria are not meant to be used to disqualify researchers from authorship credit by denying them the ability to be involved in drafting or revising the manuscript or approving the final version to be published. All researchers meeting the first criterion should be given the opportunity to participate in drafting, revising and approving the manuscript. Contributors who are unable to meet all four criteria for authorship should be acknowledged in an Acknowledgement section after obtaining their permission (ICMJE 2019)

Hence, the aim of this paper is to determine, in relation to the ICMJE authorship criteria, how authorship should be handled in situations where researchers have contributed substantively to the research and drafting of the manuscript, but the results themselves are not included in the final manuscript. Before we discuss this issue in greater detail, we would like to flesh out the problem by providing a case.

## A Case of Omitted Results

To recognize the problem we have in mind, consider the following case: Two senior researchers and three junior researchers work together on a study. One of the senior researchers take the main responsibility for conception and design of the study and assume the role of principal investigator (PI), while the other senior researcher helps substantially with suggestions and input regarding specific analyses, and also provides support in the lab, where the empirical data is obtained. The empirical work is divided among the three junior researchers Ann, Bo, and Choi in such a way that they are individually fully responsible for some part of the design and conducting the lab work, resulting in data collection, analysis and interpretation of results. As times passes, Ann, Bo, and Choi all spend many hours working hard and eventually deliver according to plan. When looking at the results and discussing the study further, all researchers on the team agree on the contents of the paper. It turns out that while the analyses made by Ann and Bo fit well with the final idea of the paper, Choi’s analyses fall outside the scope of the narrative eventually decided upon and are therefore at the end not included. This is where the discussion starts in the group. Should Choi be included as co-author? She will surely do her part in revising the manuscript and approve the final version to satisfy the second and third ICMJE criteria, but did she make a ‘substantial contribution’ to the work under the first criterion of the ICMJE recommendations? Choi has contributed to discussions throughout the life of the project at team meetings and did help in the design of her part of the experiments and conducted those studies, collected and analyzed the data, and helped interpret the results. However, she was unable to contribute to the overall conception and design of the project at this early stage of her career, similar to Ann and Bo. On an honest estimation, it can be concluded that she has not made a substantial contribution to the paper if the excluded results, her main contribution, do not make it into the final manuscript. So what to do?

Before we move on, let us notice that there are several possible reasons for not including Choi’s contribution in the paper. One reason could be that the quality of her work is too low. Another reason could be that her results are relevant but do not support the overall thesis of the paper–they are in this respect so-called negative results. As we have already indicated, omitting negative results might be problematic from a research ethics perspective, especially if the reason for omitting them is that they contradict one’s hypothesis.^1^ However, another reason for not including Choi’s contribution could be that the results are taken not to be relevant (or relevant enough) to the paper eventually decided upon, meaning that her results helped shape the final story being told in the paper but were not being reported in the publication. For the sake of argument, our discussion in what follows assumes that the reason for omitting Choi’s contribution is ethically sound and does not constitute a research misbehaviour in its own right.

## Substantial Contribution and Intellectual Involvement

As noted in the introduction, the problem we describe concerns the first ICMJE criterion for authorship. This criterion does two things: it tells us the broad categories of contributions that count towards authorship on the byline (conception or design, acquisition, analysis or interpretation of data), and it tells us the extent of contribution needed, namely that the contribution(s) have to be ‘substantial.’ For the sake of argument, let us assume that Choi’s research contributions would clearly have been substantial enough to grant a position on the paper if her data and analyses had been included in the manuscript. Now if they are not included, does it mean that Choi should not be listed as an author? And if she should, how is this compatible with the idea of a substantial contribution?

We believe that situations like Choi’s reveal that the ‘substantial contribution’ requirement of the ICMJE recommendations is not only vague in terms of what is required for a contribution to be large enough to be *substantial* (Cutas & Shaw, 2015; Laflin et al. 2005; Osborne & Holland, 2009), but also ambiguous in terms of what *specifically* counts as a contribution. In particular, we hold that we should distinguish between two interpretations of this criterion, namely whether it concerns a substantial contribution to *what ends up in the paper* or whether it concerns a substantial contribution to *the research process leading up to the paper*. While Choi does not qualify as an author in the former sense, she might do so if we accept the latter interpretation of the substantial contribution criterion. The question remains which of the two interpretations of ‘substantial contribution’ is the most plausible one.

To be certain, making a substantial contribution is not enough to qualify for authorship according to the ICMJE criteria. Critical revision and final approval of the manuscript outlined in the second and third ICMJE criteria is needed as well. The critical revision requires intellectual involvement in the paper under production. Hence, intellectual involvement in the research at hand is part of the ICMJE authorship requirements (Helgesson, 2015).

Before returning to our case, let us first present and consider another one in which the main point is to clearly show that one might make a substantial contribution to the research of a paper without contributing to what ends up in the paper. Assume that a group is writing a paper on research methods for accomplishing *X* in the research field of *Z*. They proceed by examining all existing methods mentioned in the literature potentially relevant for the specific purpose at hand, dividing the work among them so that each researcher involved analyzes the same number of methods each. The results from the analyses of the methods found to work are described at some length in the final paper, while failing methods are merely mentioned (although they are as completely documented by the research group). Since it is not known beforehand which methods will work and which ones will not, each analysis will be equally relevant to the fulfillment of the aim of the paper–to clarify the best methods to accomplish *X*. If the research contribution is substantial, and other authorship criteria are fulfilled, all researchers should be included as co-authors of the paper irrespective of whether their method is presented in the manuscript or not. Hence, there is a case to be made that substantial research contributions sometimes should count towards authorship even if they are not represented equally in the final paper.

## Fairness, Transparency and Accountability

As previously detailed, we believe that cases like Choi’s reveal an ambiguity concerning the notion of ‘substantial contribution.’ It might be understood as saying that what is required is either a substantial contribution to what ends up in the paper or a substantial contribution to the research leading up to the paper. If the first interpretation is correct, then Choi should be excluded from authorship, despite the work and effort that she has put into this collaborative work. If Choi ought to be included in the paper, which we think she should, this is because it is enough, with respect to the first authorship criterion, that she has contributed in a substantial and relevant way to the research leading up to the paper, even if her specific empirical contributions did not end up in the paper. We will therefore defend this interpretation of the substantial contribution requirement of the ICMJE recommendations.

Generally, there are two sets of reasons for caring about how authorship is handled when a researcher makes a substantial contribution that is not reported in the manuscript. First, it is a matter of transparency and accountability about the research process: what happened and who were involved? From this perspective, it is misleading if people who were deeply involved in the work are not described in the paper. Also for reasons of accountability, those responsible for the work should be identifiable to others. Admittedly, both of these aspects could be fulfilled by some other means than that of attribution of authorship, such as a sufficiently detailed description of everyone’s contributions, including those not included as authors. But with present practices, including someone as co-author or merely listing that person in the contributor list or acknowledgements communicates two quite different messages about the person’s contribution and its relative importance. Our argument here for the interpretation that what counts as ground for co-authorship is a substantial contribution to the research leading to the paper is that excluding Choi would be misleading about her role in the project, hence not fulfilling the need for transparency and accountability.

Second, authorship is a matter of due scientific credit and fairness, which to our mind provides a strong reason for including Choi. In particular, our argument for the interpretation that what counts is a substantial contribution to the research leading to the paper is that excluding Choi seems unfair. After all, Choi has contributed as much to the research leading to the paper as her colleagues. Admittedly, the aim of the paper shifted, or took a form that made Choi’s contribution less relevant to what was reported in the paper, but not less relevant to the end product leading to the research publication. Also, we should note that this interpretation clearly ties Choi’s claim to authorship to the good work she has done rather than to the results being reported only. In contrast, if what is required is a substantial contribution to the research *presented* in the paper, then it seems that whether any of the junior researchers end up in the authorship byline will be, to some degree, a *matter of luck*, since neither an initial agreement or great efforts along the way is sufficient. This too seems unfair. Contributing with a considerable amount of good work and then not receiving authorship because of a change of plans seems unfair in a vein similar to misuse of the ICMJE authorship criteria by letting people contribute substantially to the work, then not offer them the opportunity to revise, and finally not include them as authors since they do not fulfill all criteria (ICMJE, 2019).

## Counter Arguments

There are several possible counterarguments to our thesis that should be addressed. First, it might be argued that Choi’s contribution is rather general in its nature, and hence should not count towards authorship. After all, authorship is not about general research contributions, such as having contributed substantially to a large research application providing financial support for the production of many papers (reaching a level of detail that the application did not), but contributions to *the specific paper* (Smith & Master, 2017). Similarly, being a member of the research group does not mean that one should be included as co-author on every paper produced by the group. Instead, you need to contribute substantially to every paper in order to be listed as author. However, one may agree with the general thrust of this argument without agreeing that it works as an argument against including Choi. That is because it is an open question what should be meant by ‘contributions to the specific paper’–is it what the final version of the paper contains or is it the work specifically concerning and leading up to that paper? It seems to us that the better understanding of ‘research contribution’ is the work and intellectual engagement contributed in the context of a study or project around a specified research aim and/or a set of specified research questions, rather than what of that work was eventually included in the paper–as long as the work, as it was carried out, was perceived by the research group as relevant to what they were doing.

Second, one might ask why it would not be enough if Choi’s contribution were recognized through an acknowledgement, especially if it contains a clear statement of her contribution. Important contributions that do not qualify for authorship are often handled that way, so why shouldn’t we think that it is enough in this case? There are two connected responses to this inquiry. First, as already argued, Choi has made contributions qualifying her for authorship. Leaving her out would therefore be misleading in the sense that it would give a false impression about the relative value of her contribution to the research. Second, it would be utterly unfair to grant her an acknowledgement if her colleagues, making contributions of equal importance (according to our reasoning above), are included as authors. Again, as discussed above, allocation of authorship concerns transparency and accountability on the one hand, and credit and fairness on the other. Acknowledgements contribute to transparency, but are useless from a career perspective in most, if not all, research areas, hence leaving Choi with an inappropriate credit for her work.^2^

Third, there is a complication with our interpretation of the substantial contribution requirement that we may call the *multiplicity problem*. Imagine that the overall thrust of our argument was followed by the research group and that Choi was included as co-author in the paper based on her substantial contributions to the work with the paper even though her work was not presented in the paper. Further imagine that later on, the senior researchers find it a good idea to include the previously excluded work by Choi in another paper where it becomes more relevant. The work then included is indeed substantial, which provides an argument why Choi should be included as co-author also in this second paper. But doesn’t this amount to an unfair form of double counting? In response, we think that it might be acceptable to include Choi as co-author on both papers, insofar as she fulfills the following two conditions: the contribution under the first criterion has to be different in the two papers (which is not the same thing as saying that the contribution has to concern different data sets), and the other ICMJE authorship criteria need to be fulfilled in relation to the second paper as well. Admittedly, Choi was not part of the research process, and perhaps not intellectually involved in the questions particularly addressed in this second paper, at an early stage. But once included with her empirical contribution (originally omitted and hence different from her contribution to the first paper), she could engage in the larger questions of this second paper and could contribute intellectually as well. For example, she could participate in the process of revising versions of the manuscript critically for important intellectual content, as put in the second criterion of the ICMJE authorship criteria. If so, then it seems right that she is included as an author also in the second paper since she fulfils criteria 1–4 in relation to that paper.^3^

But then there seems to be a further complication with our account that we might call the *backfire problem* (which is a counter argument to our response to the multiplicity problem). Again, assume that Choi was included as an author on the first paper where her work was not presented in the paper itself, but that the work has also begun with the second paper based on Choi’s contribution. Ann and Bo now enter the scene and ask why they are not included in the second paper–should they be? After all, if Choi ought to be included as an author on the first paper, on the basis that her work with the omitted empirical contribution was an important part of the research process leading up to the first paper, then doesn’t this suggest that Ann and Bo can raise similar legitimate demands on being included as authors on the second paper? In response, we suggest that the answer can certainly not be a general ‘Yes,’ but that their inclusion in the second paper is justifiable if they were sufficiently involved in the second paper to be correctly described as having contributed substantially to the process leading up to *that* specific paper. For example, the second paper might be a natural follow-up of the first paper, covering further issues already considered while they were working on the first paper.^4^ However, it all hinges on whether Ann and Bo contributed substantially, in the sense we have defended in the above.

Before concluding we should make two important remarks. First, our arguments in response to the *multiplicity problem* has important implications beyond the case that we are focusing on here. For example, when a biomedical researcher builds a new reagent and publishes it, the original creator does not (or at least should not) receive authorship recognition each and every time the reagent is shared and used by other scientists in subsequent studies. The reason for this, or so we would argue, is that merely providing certain substances or data is not enough to qualify as an author. What is important is rather that one has contributed substantially in the research process leading up to the paper. However, allowing people to use one’s innovation over and over does not guarantee the type of involvement required for authorship in a specific paper. Second, the focus of this paper has only been on authorship attribution, but cases like the one we discuss here also raise issues about authorship order, which is a contentious area with as yet no uniform policy or practice which guides researchers (Helgesson & Eriksson, 2019; Smith & Master, 2017). Addressing this issue is, however, beyond the scope of this paper.

## Concluding Remarks

In this paper we have argued that authorship should be attributed to those who have made a substantial contribution to the specific research leading up to the publication, even if their particular contribution is not reported in the paper. Based on the principle of fairness and giving credit where credit is due, researchers who make significant contributions should be given authorship credit even if their contribution is not included in the final manuscript. We have argued that this practice is aligned with the most plausible interpretation of the first criterion of the ICMJE recommendations, and thus researchers should be afforded the opportunity to participate in fulfilling subsequent criteria including drafting or critically revising the manuscript, approving the final version of the paper, and agreeing to be accountable for the research. Based on our analysis, we suggest that ICMJE and others interested in authorship and publication ethics need to revise their proposals regarding authorship allocation for the sake of clarity.

As a suggestion for future research, further conceptual and empirical work in this area should examine and consider situations where substantive but excluded contributions deserve or not deserve authorship credit. While several studies examine gift authorship (e.g., Sauermann & Haeussler, 2017; Wislar et al 2011), less has been done to assess the nature and frequency of authorship exclusion, which is likely to be significant and affect those with less power in academic science (Cesi and Williams 2011; NASEM 2017).

## Data Availability

Not applicable.

## Footnotes

1 There is much more to be said here. The handling of negative results has been discussed extensively in the research ethics literature, mainly in relation to selection bias when it comes to what studies get published and not. There are ethical issues involved here, relating to reasons for not publishing, such as researchers being misleading by withholding results, wasted resources, and introduction of bias into meta-analyses. The tendency to favor positive results is systemic (Chalmers, 1990; Duyx et al., 2017; Dwan et al., 2008; Fanelli, 2012; Song et al., 2000). Addressing these sorts of issues is beyond the scope of this paper, however, as our focus is to analyze the ethics of who should be included as authors in cases where not all work done has explicit impact on what ends up in print.

2 The fact that acknowledgements do not carry any weight from a career perspective might be a reason for ditching both acknowledgements and traditional author lists in favour of more detailed contributorship statements at the beginning of academic papers. Such a system would at best not only be more transparent, but also more accurate when it comes to the allocation of academic credit. That said, our argument here largely applies to the present situation, in which authorship is the norm.

3 We admit that our solution to the multiplicity problem still leaves open for disputes over authorship. After all, researchers might still disagree whether a particular contribution is sufficiently different from another one. That said, we still believe that what we say in response to the multiplicity problem is valid and should be adopted as a general starting point for how to handle this kind of disputes. Thus, we suggest that the principle is valid, even if the application of the principle can still be debated in individual cases.

4 We want to underline that we do not suggest that so-called salami-slicing should be acceptable. However, you do not get salami slicing just because you make more than one paper out of a data set. Salami slicing is if you separate your data into several papers merely in order to get more papers, not because it is justified for scientific considerations, such as making the content comprehensible and the story sufficiently clear for the reader to follow (not to mention managing limitations of space).
